# Racial Bias in Neural Response for Pain Is Modulated by Minimal Group

**DOI:** 10.3389/fnhum.2017.00661

**Published:** 2018-01-11

**Authors:** Fengtao Shen, Yang Hu, Mingxia Fan, Huimin Wang, Zhaoxin Wang

**Affiliations:** ^1^Key Laboratory of Brain Functional Genomics, Ministry of Education, Shanghai Key Laboratory of Brain Functional Genomics, Institute of Cognitive Neuroscience, School of Psychology and Cognitive Science, East China Normal University, Shanghai, China; ^2^Shanghai Key Laboratory of Magnetic Resonance, East China Normal University, Shanghai, China; ^3^New York University-East China Normal University Institute of Brain and Cognitive Science, NYU Shanghai, Shanghai, China

**Keywords:** racial bias, ingroup bias, minimal group, empathy, implicit racial bias

## Abstract

Whether empathic racial bias could be modulated is a subject of intense interest. The present study was carried out to explore whether empathic racial bias for pain is modulated by minimal group. Chinese/Western faces with neutral expressions receiving painful (needle penetration) or non-painful (Q-tip touch) stimulation were presented. Participants were asked to rate the pain intensity felt by Chinese/Western models of ingroup/outgroup members. Their implicit racial bias were also measured. Two lines of evidence indicated that the anterior cingulate cortex (ACC) was modulated by racial bias: (1) Chinese models elicited stronger activity than Western did in the ACC, and (2) activity in the ACC was modulated by implicit racial bias. Whereas the right anterior insula (rAI) were modulated by ingroup bias, in which ingroup member elicited stronger activity than outgroup member did. Furthermore, activity in the ACC was modulated by activity of rAI (i.e., ingroup bias) in the pain condition, while activity in the rAI was modulated by activity of ACC (i.e., racial bias) in the nopain condition. Our results provide evidence that there are different neural correlates for racial bias and ingroup bias, and neural racial bias for pain can be modulated by minimal group.

## Introduction

Empathy refers to the capacity to understand and respond to the unique affective experiences of other person (Decety and Jackson, 2006), which is believed to be a key motivator and the proximate mechanism of altruistic and prosocial behavior (Batson et al., 1991; Preston and de Waal, 2002; Singer et al., 2006; Moriguchi et al., 2007). The majority of studies using functional magnetic imaging (fMRI) to unveil the neural mechanisms of human empathy used paradigms in which participants were exposed to stimuli depicting or indicating that other people were in pain (vs. no pain), one of the most basic and universal human experiences (Lamm et al., 2011). A meta-analysis indicated that a core network consisting of bilateral anterior insular cortices and medial/anterior cingulate cortex is associated with empathy for pain (Lamm et al., 2011). Importantly, empathic neural responses for pain in this network predict individual differences in costly helping (Hein et al., 2010), which is in line with the suggestion that empathy is related to prosocial behavior. Interestingly, a number of recent studies on empathy for pain have provided evidence of an empathic bias toward own race members (Xu et al., 2009; Avenanti et al., 2010; Contreras-Huerta et al., 2013). It was also found that empathic sensorimotor responses for pain could be modulated by implicit racial bias (Avenanti et al., 2009, 2010). Indeed, racism often manifests itself as a lack of empathy for other-race (Bell, 1980). Whether racial empathic bias can be reduced by a simple way is an open question.

It has been recently demonstrated that response for pain depends on the social relationships between the observer and the individuals experiencing the outcome (Montalan et al., 2012). For example, Hein et al. (2010) reported that soccer fans display stronger empathic neural responses in the anterior insula (AI) while witnessing a fan of their favorite team (ingroup) experience pain (vs. a fan of a rival team, or outgroup). Following this idea, an ERP study showed that including other-race individuals in one’s own team for competitions increases neural responses to facial pain expressions in other-race faces (Sheng and Han, 2012). It should be noted that the competition between groups can generate undesired negative social implications, such as hostility to outgroup (Gaertner and Dovidio, 2000). Tajfel reported that the mere categorization of individuals into two social groups on the basis of arbitrary criteria, i.e., a minimal group paradigm, is sufficient to produce ingroup bias as compared to natural groups without eliciting explicit negative affective factors (Tajfel, 1970). It has been found that a minimal group paradigm may diminish automatic racial bias (Kurzban et al., 2001; Van Bavel and Cunningham, 2009). Moreover, a behavioral experiment showed that the mere categorization of adults on the basis of minimal criteria is sufficient to elicit an ingroup bias in empathy for pain (Montalan et al., 2012). Similarly, children also display more empathy bias favoring their ingroup after a week’s novel group merely based on color (Masten et al., 2010). Thus, a minimal group paradigm may be used to reduce empathic racial bias. However, to our knowledge, the only two imaging studies (one fMRI study and one ERP study) failed to detect any significant effect of intergroup relationships on racial bias in empathic neural responses for pain (Contreras-Huerta et al., 2013, 2014). These findings led to the first concern that the possible effects of minimal group on racial empathic bias, if exist, are very weak. Further study using a more sensitive approach is required to clarify this question. Regions of interest (ROI) approach yields higher sensitivity than whole brain analyses (Mitsis et al., 2008) and thus is used in the present study.

A second concern is that typically the contrast of pain and nopain conditions is considered neural empathic response for pain in most studies (Lamm et al., 2011). However, participants with stronger implicit racial bias show increased BOLD signal to other race’s faces (i.e., a nopain condition) (Phelps et al., 2000). On the contrary, participants with stronger implicit racial bias show decreased empathic sensorimotor resonance (the contrast of pain vs. nopain conditions) to other race’s hand model (Avenanti et al., 2010). Obviously, these opposite patterns suggest that neural responses to race might be different between nopain and pain conditions. If so, mathematically the decreased empathic responses (contrasts of pain vs. nopain) related to stronger racial bias might not necessarily reflect the decreased neural responses for other’s pain, but just represent the increased neural responses in the nopain condition. Also, there the opposite patterns between nopain and pain conditions should lead to larger individual differences in the contrasts of pain vs. nopain, which makes it even harder to detect the possible weak effects of minimal group on racial bias. Thus, it would be helpful to analyze the data of pain and nopain conditions separately to address this concern.

In the present study, we investigated whether racial bias in neural response for pain is modulated by minimal group with a relative larger sample (29 participants). A non-competitive mix race minimal group paradigm was adopted, in which all participants were randomly assigned to a novel group. They were asked to memorize their in- and outgroup faces through several minutes’ learning and memory (Kurzban et al., 2001; Van Bavel et al., 2008). Later, they were asked to rate the pain intensity of these faces with neutral expressions receiving painful (needle penetration) or non-painful (Q-tip touch) stimulation were presented during fMRI scanning (Xu et al., 2009). Novel Chinese and Western faces with matched attractiveness were used to rule out historical events as well as personal involvement that may confound the mechanism of simple group categorization. An ROI approach was applied, as this approach yields higher sensitivity than whole brain analyses (Mitsis et al., 2008). Participants’ implicit and explicit attitudes toward Chinese and Westerns were also measured. We hypothesized that (1) neural responses can be modulated differently by minimal group and race, (2) neural responses are differently modulated by implicit racial bias, and (3) racial bias in neural response for pain can be modulated by minimal group.

## Materials and Methods

### Participants

A total of 37 Chinese students from East China Normal University (6 males; mean age = 20.6 years, *SD* = 1.8) voluntarily attended the current study. Data from 8 participants attending the pilot study was not analyzed, and the remaining 29 participants were used for further analysis (4 males; 14 in Red Group and 15 in Green Group; mean age = 20.5 years, *SD* = 1.7). All were right-handed with normal or corrected-to-normal vision and normal color perception, and reported no psychiatric or neurological history. After completing all tasks, participants were debriefed and paid as compensation for their time. Written informed consents were obtained from all subjects, and the protocol was approved by the University Committee on Human Research Protection (UCHRP) at East China Normal University.

### Materials and Procedure

#### Stimuli

**FIGURE 1:**
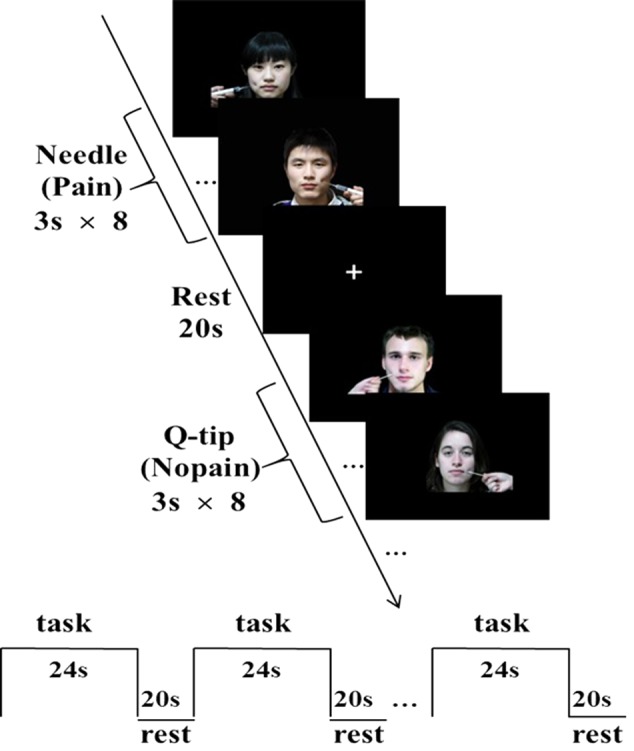
Sample stimuli and fMRI experimental design. A block design was applied. Each block consisted of eight trials. Each 3-s trial depicted either ingroup or outgroup members from the same or difference race receiving painful (needle) or non-painful (Q-tip) stimulation, applied in either the left or the right cheeks.

A total of 16 novel colorful photographs of face with neural expression were used. They were divided into two groups: one was assigned as ingroup members and the other as outgroup members to participants pseudorandomly. Each group consisted of eight different faces including four Chinese faces (two males) and four Western faces (two males, all Caucasian), respectively. Facial attractiveness was matched according to subjective rating of a separate group of naïve participants (*n* = 28). Each participant was also asked to provide a standard digital photograph of his/her own face with neutral expression and the photo, after processed to fit experimental standard, was included in his/her learning task (but not in the MRI session) to enhance group identification (Van Bavel et al., 2008). These faces with neutral expressions receiving painful (needle penetration) or non-painful (Q-tip touch) stimulation applied in the left/right cheeks were then used in the fMRI session (**Figure 1**). The mean luminance of the Western faces (85.4, *SD* = 11.9) is higher than that of Chinese faces (70.3, *SD* = 12.0, *t*_62_ = 5, *p* = 0.000004), while the contrast values are not significantly different between races (4.7 ± 0.6 for Western faces and 4.5 ± 0.7 for Chinese faces, *t*_62_ = 1.1, *p* = 0.28). The contrast is calculated by the following equation:

$$\begin{array}{c} \mathrm{Contrast}\;\mathrm{Value}=\frac{\mathrm{Face}\;\mathrm{Luminance}\;-\;\mathrm{Background}\;\mathrm{Luminance}}{\mathrm{Overall}\;\mathrm{Luminance}} \end{array}$$

#### Pre-scanning Group Assignment

Having arrived at the imaging center, participants were informed that the current study aimed to explore whether they could integrate into a novel minimal group as soon as possible, in which members were with multi-cultural backgrounds. They were pseudorandomly assigned to a Red Group (*n* = 19) or Green Group (*n* = 18), and were told to distinguish their ingroup members from outgroup members through the following learning task. The whole learning task lasted for about 15 min.

During the first learning task, participants were instructed to memorize to which group these members belong. In each trial, a face was presented for 3 s, accompanied with a red or green frame to indicate group membership, with a 1-s inter-trial fixation cross. Faces from the same group were presented sequentially in a block manner and the order of group block was counterbalanced. Each face repeated four times during the whole phase.

The second learning task consisted of two blocks. In the first block, the procedure of each trial was the same as that of the first learning task except that participants were instructed to categorize each face in terms of their group membership within 3 s by pressing either “F” (ingroup members or him-/herself) or “J” (outgroup members). Then, a cartoon animation depicting either “pass a ball” or “hold a ball” for in-/outgroup members, respectively, was shown, to enhance participants’ involvement. The second block was a category task, which was the same as the first block, with the exceptions as follows: (1) the color frame was removed, so that participants had to judge the group membership of each face relying on their memory; (2) each trial was followed by a feedback indicating whether the response was correct. A wrong response would lead to another judgment to the same stimuli again. A total of 30 participants with accuracy higher than 90% in this block were qualified to enter the MRI session.

#### fMRI Task

A block design was applied. There were four functional runs in total. Each run consisted of eight blocks with a 2 × 2 × 2 design, each block depicted either ingroup or outgroup members from the same or difference race receiving painful or non-painful stimulation. Each block consisted of eight trials, with four different faces of the same race (four males) with neutral expression receiving painful or non-painful stimulation, applied in either the left or the right cheeks. In each trial, a stimulus without any cue of group membership was presented for 3 s, during which time participants were asked to rate the pain intensity felt by the model using a hand-shaped response box with their right hands, ranging from “1 = not painful at all” (with little finger) to “5 = severely painful” (with thumb) with each one corresponding to one finger. There was a 20-s inter-block interval, with a white fixation cross in the middle of the screen. The order of blocks within these four functional runs was counterbalanced. Stimuli were presented through goggles system (InVivo Co., United States).

#### Post-scanning Procedure

Participants were first instructed to categorize each face again in terms of their group membership within 3 s to test whether they have memorized ingroup and outgroup members. Three questionnaires were used to assess (1) empathic ability, assessed by the Interpersonal Reactivity Index (IRI) (Davis, 1983), (2) attitudes of ethnic identity, measured by the Multigroup Ethnic Identity Measure (MEIM) (Phinney, 1992), and (3) the degree of agreement of individualistic and collectivistic values, detected by the Horizontal and Vertical Individualism and Collectivism (HVIC) scale (Triandis, 1996).

#### Racial Attitudes Measurement

Participants’ implicit and explicit attitude toward Chinese and Westerners were measured on a separate day after the fMRI session. The implicit racial attitude was measured by the Implicit Association Test (IAT) (Greenwald et al., 1998, 2003) using novel neutral Chinese and Western faces with matched attractiveness (Avenanti et al., 2010). D-index of each participant was calculated (Greenwald et al., 2003). The explicit attitude was measured by a 5-point Likert scale (1–5) and a 11-point thermometer scale (0–10) on two racial groups (Greenwald et al., 2003).

### Image Acquisition

The scanning was conducted on a 3-Tesla Siemens Trio MR scanner, including four functional runs and one anatomical run in total. For functional images, 35 axial slices (FOV = 240 mm × 240 mm, matrix = 64 × 64, in-plane resolution = 3.75 mm × 3.75 mm, thickness = 4 mm, without gap) covering the whole brain were obtained using a T2^∗^-weighted echo planar imaging (EPI) sequence (TR = 2000 ms, TE = 30 ms, flip angle = 90°). A high-resolution structural image was also acquired using 3D MRI sequences for anatomical co-registration and normalization (TR = 1900 ms, TE = 3.43 ms, flip angle = 7°, matrix = 256 × 256, FOV = 240 mm × 240 mm, slice thickness = 1 mm).

### Data Analysis

#### Voxel-Wise Whole Brain Analysis

SPM8^1^ was adopted for fMRI data analysis (Wellcome Department of Cognitive Neurology, London, United Kingdom). For each subject, EPI images were first realigned to the first volume to correct for head motion. One subject was excluded from further data analysis because of excessive inter-run head motion (> 2 mm). Then, the anatomical image was co-registered with the mean EPI image, which was further segmented and then generated normalized parameters to MNI spaces. Next, all EPI data were projected to MNI template with a re-sampled voxel size of 2 mm × 2 mm × 2 mm. Finally, the functional images were spatially smoothed with a Gaussian kernel with a full width at half maximum (FWHM) of 8 mm. To remove low-frequency drifts, high-pass temporal filtering with a cutoff of 128 s was carried out.

Data from 29 participants were used for further analysis. For the first-level analysis, a boxcar model with eight conditions convolved with the canonical hemodynamic responses embedded in SPM (HRF) was applied. The six estimated head movement parameters were included in the design matrix to remove residual effects of head motion. Parameter estimates were then subject to a second-level analysis, a 2 × 2 × 2 ANOVA with condition (pain vs. nopain), race (Chinese vs. Western), and group (ingroup vs. outgroup) as factors with participants as dependent factors. The voxel-wise threshold was set at *p* = 0.005. To evaluate brain activations of empathy-related brain regions, the activation map was then masked by the contrasts between pain/nopain condition and rest as well as the contrast between pain condition and nopain condition (*p* < 0.005, uncorrected; *k* = 30). A Small Volume Correction was used in a sphere with radius = 8 mm.

#### Region-of-Interest (ROI) Analysis

An ROI approach was then performed on regions survived in the voxel-wise analysis (Wang et al., 2010). Parameter estimates of signal intensity of 29 participants within each ROI were extracted using AFNI software package (Cox, 1996) for further repeated-measures ANOVA analysis, and paired *t*-test as *post hoc* analysis, threshold was set to *p* = 0.05 (one-tailed) for regions we have prior hypothesis, i.e., the AIs, in which ingroup members are expected to elicit stronger brain activity than outgroup members do.

We also calculated correlations between fMRI data of nopain/pain vs. rest and dispositional measures. We further assessed the relationship between the BOLD signals of the ACC and that of the rAI using correlation analysis. Bonferroni correction was used to correct for multiple comparison.

## Results

### Dispositional Measures

Dispositional measures were listed as mean (SD). The mean score of IRI was 69.5 (9.3) with the mean of the empathic concern subscale was 19.0 (4.6). The mean score of MEIM was 56.7 (8.5). For the measurement of HVIC, no significant difference was found between the scores of Collectivism subscale and Individualism subscale [6.8 (1.0) and 6.5 (0.8), respectively; *t*_28_ = 1.4, *p* = 0.18]. All these scores were within the range of the norm values.

For the implicit racial attitude, the results of the IAT showed that D-index was significantly greater than zero (*t*_28_ = 4.6, *p* < 0.001), indicating an implicit preference for own-race to other-race group members. For the explicit racial attitude, participants preferred Chinese people to Western people [3.5 (0.8); *t*_28_ = 3.1, *p* = 0.005] and showed more positive attitudes toward own-race people than other-race people [Chinese: 7.6 (1.1); Western: 6.4 (1.2); *t*_28_ = 3.7, *p* = 0.001].

### Behavioral Results

Neither main effect nor interaction on response accuracy toward in- or outgroup Chinese/Western faces (*F*s_1,28_ < 1.2, *p*s > 0.1) was found between pre- and post-scanning categorization tests. The average response accuracy of 29 participants in categorization task was shown in **Table 1**.

**Table 1:** Mean response accuracy (SD) in pre- and post-scanning categorization test (*N* = 29).

	Chinese		Western		Total	
	Ingroup	Outgroup	Ingroup	Outgroup	Ingroup	Outgroup
Pre-scanning (%)	97.7 (4.8)	97.1 (4.4)	98.7 (3.5)	98.3 (4.0)	98.1 (3.1)	97.7 (3.0)
Post-scanning (%)	99.0 (2.6)	98.5 (3.9)	98.5 (2.5)	99.0 (4.2)	98.6 (2.2)	98.9 (2.4)

In the fMRI session, faces penetrated by a needle were rated more painful than those touched by a Q-tip (*F*_1,27_= 167, *p* < 0.001). Furthermore, participants displayed higher rating scores toward Chinese faces in comparison with Western faces (*F*_1,27_= 49.0, *p* < 0.001) both in the nopain and pain condition (*t*s > 5.1, *p*s < 0.001). Regarding the reaction time, both main race effect and race × condition interaction were found (*F*s > 25.9, *p*s < 0.001), and these differences came from the Q-tip condition of the Western faces, which was much faster than any other conditions (*t*s > 7.0, *p*s < 0.001). No other main effect or interaction was found (*F*s_1,27_< 3.9, *p*s > 0.06). Please see **Table 2** for details.

**Table 2:** Mean rating scores (SD) of pain intensity and reaction time (RT) during fMRI scanning (*N* = 28).

	Pain condition				Nopain condition			
	Chinese		Western		Chinese		Western	
	Ingroup	Outgroup	Ingroup	Outgroup	Ingroup	Outgroup	Ingroup	Outgroup
Rating	2.9 (0.7)	2.8 (0.7)	2.7 (0.8)	2.6 (0.7)	0.8 (0.5)	0.8 (0.5)	0.4 (0.5)	0.4 (0.3)
RT (ms)	1251 (226)	1244 (232)	1287 (245)	1311 (230)	1234 (227)	1248 (225)	1103 (205)	1097 (196)

*Data from one subject was lost due to technical reasons.*

### Imaging Results

**FIGURE 2:**
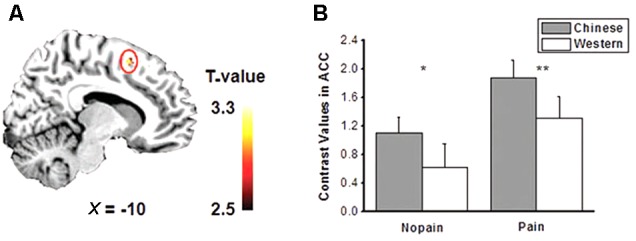
**(A)** Activity of anterior cingulate cortex (ACC) was modulated by race (*p* < 0.005, small volume corrected). **(B)** Chinese models elicited stronger ACC activity than that of Western models both in the pain and nopain conditions (SE). ^∗^*p* < 0.05, ^∗∗^*p* < 0.02, one-tailed.

**FIGURE 3:**
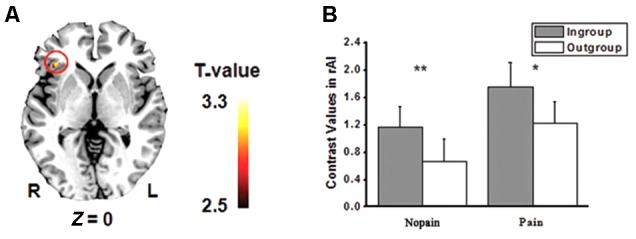
**(A)** Activity of right anterior insula (rAI) was modulated by minimal group (*p* < 0.005, small volume corrected). **(B)** Ingroup models elicited stronger rAI activity than that of Outgroup models both in the pain and in the nopain conditions (SE). ^∗^*p* = 0.05, ^∗∗^*p* < 0.02, one-tailed.

The main effects of empathic responses for pain (i.e., contrast of pain vs. nopain) were found in the ACC and bilateral AIs, where participants showed stronger activity for the pain condition than the nopain condition, in line with previous findings (Lamm et al., 2011). In these brain regions, the main effect of race was found in the ACC (**Figure 2A**), where participants displayed increased activity to Chinese members than Western members (*F*s_1,28_ > 7.9, *p*s < 0.01) both in the nopain condition and in the pain condition (*t*s_28_ > 2.2, *p*s < 0.05, **Figure 2B**). The main effect of group was found in the right AI (**Figure 3A**), where participants showed increased responses toward ingroup members than outgroup members (*F*s_1,28_ > 7.9, *p*s < 0.01) both in the nopain condition and in the pain condition (*t*s_28_ > 1.7, *p*s ≤ 0.05, **Figure 3B**). Participants also displayed increased responses toward ingroup members than outgroup members in the orbitofrontal cortex (Small Volume Correction), whereas increased responses toward Western members than Chinese were observed in the visual cortices (FWE corrected). No significant interaction was found.

**FIGURE 4:**
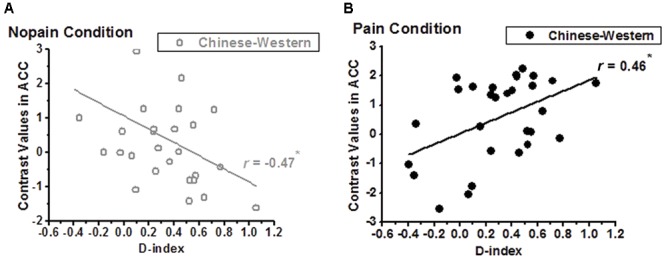
Correlation between D-index and the contrasts of Chinese vs. Western of the ACC. **(A)** a significant negative correlation was found in the nopain condition (indexed by gray), but **(B)** a significant positive correlation was found in the pain condition (indexed by black), ^∗^adjusted *p* < 0.05.

We calculated correlations between contrast values of nopain/pain vs. rest and dispositional measures, and found a negative correlation between D-index and racial bias (i.e., contrast of own-race vs. other-race) of ACC of non-painful stimuli vs. rest (*r* = -0.47, adjusted *p* = 0.022 after Bonferroni correction, **Figure 4A**) but a significant positive correlation between D-index and racial bias of ACC of painful stimuli vs. rest (*r* = 0.46, adjusted *p* = 0.025, **Figure 4B**). *Post hoc T*-test further showed a significant difference between the correlation coefficients in the pain condition and that in the nopain condition (*t*_26_ = 4.54, *p* < 0.001).

**FIGURE 5:**
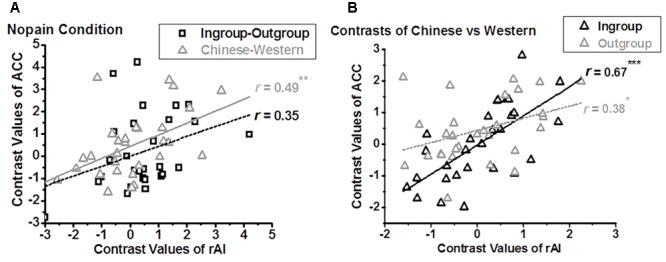
Neural activity of the rAI is modulated by racial bias in nopain condition **(A)**, both for ingroup and outgroup **(B)**. In the nopain condition, a positive correlation is found between the contrasts of Chinese vs. Western (i.e., racial bias) of ACC and that of rAI (**A**, triangle; ^∗∗^adjusted *p* < 0.05), both for Ingroup and Outgroup members (**B**, ^∗^*p* < 0.05, ^∗∗∗^*p* < 0.001, two tailed), but not between the contrasts of Ingroup vs. Outgroup (i.e., ingroup bias) of ACC and that of rAI (**A**, rectangle).

**FIGURE 6:**
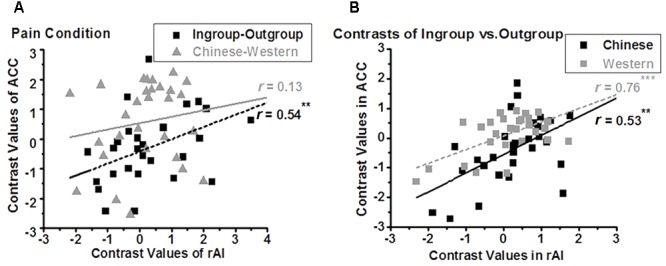
Neural activity of ACC is modulated by ingroup bias in pain condition **(A)**, both for Chinese and Western **(B)**. In the pain condition, a positive correlation was found between the contrasts of Ingroup vs. Outgroup (i.e., ingroup bias) of rAI and that of ACC (**A**, rectangle, ^∗∗^adjusted *p* < 0.01), both for Chinese and Western pictures (**B**, ^∗∗^*p* = 0.003, ^∗∗∗^*p* < 0.001, two tailed), but not between the contrasts of Chinese vs. Western (i.e., racial bias) of ACC and that of rAI (**A**, triangle).

We further assessed the relationship between the BOLD signals of the ACC and that of the rAI. In the nopain condition, we found a significant positive correlation between the contrasts of own-race vs. other-race (i.e., racial bias; *r*_28_ = 0.49, adjusted *p* = 0.028 after Bonferroni correction) but not between the contrasts of ingroup vs. outgroup (i.e., ingroup bias; *r*_28_ = 0.35, *p* = 0.07,) in these two regions (**Figure 5A**), which was stood for both ingroup (*r*_28_ = 0.67, *p* < 0.001) and outgroup members (*r*_28_ = 0.38, *p* = 0.04, **Figure 5B**). In the pain condition, on the contrary, we found a significant correlation between the contrasts of ingroup vs. outgroup (i.e., ingroup bias; *r*_28_ = 0.57, adjusted *p* = 0.004) but not between the contrasts of own-race vs. other-race (i.e., racial bias; *r*_28_ = 0.17, *p* = 0.39) in these two regions (**Figure 6A**), and the correlations stood both for Chinese members (*r*_28_ = 0.53, *p* = 0.003) and Western members (*r*_28_ = 0.76, *p* < 0.001; **Figure 6B**).

## Discussion

In the present study, we investigated whether racial bias in neural response for pain is modulated by minimal group. First, we found that different brain regions were modulated by racial bias and ingroup bias. To be specific, the ACC were more activated by the faces of own-race members than that of other-race members. Beyond previous studies (Xu et al., 2009), we further found that activity of the ACC was modulated by implicit racial bias, but differently between nopain and pain conditions. Moreover, in line with our prior hypothesis, we found that the rAI was more activated by the faces of ingroup members than that of outgroup members. Second and critically, we found for the first time that neural responses of the ACC were modulated by ingroup bias in the pain condition, while neural responses of the rAI were modulated by racial bias in the nopain condition.

### Different Neural Correlates of Racial Bias and Ingroup Bias

We found that Chinese models elicited stronger activation than Western models did in the ACC, both for nopain and for pain conditions. Consistently, Xu et al. (2009) reported that the empathic neural response in the ACC decreased significantly when participants viewed faces of other races. Stanley et al. (2008) also suggest that the ACC is involved in the detection and regulation, respectively, of implicit attitudes. Our results confirmed Stanley’s suggestion in that activity in the ACC was modulated by IAT. Moreover, we found that the activity of the ACC was modulated by implicit racial bias differently in the pain condition and nopain condition. To be specific, we found a positive correlation in the pain condition, but a negative correlation in the nopain condition, between racial contrasts of the ACC and D-index. Combined, these results suggest that the ACC is involved in empathic racial bias.

On the other hand, we found neural responses in the AI, but not the ACC, were modulated by minimal group, in line with a previous study (Lamm et al., 2007), as participants showed stronger BOLD signals to ingroup members than to outgroup members in the rAI. We assume that the difference between the BOLD signals is due to neither stimulus features nor affect, because all faces were neutral and were counterbalanced between participants. In addition, there was no explicit between-group affective or competitive difference during minimal group assignment. The difference between BOLD signals comes from minimal group assignment that is just a simple separation between ‘us’ and ‘them.’ Our results significantly extend previous studies about ingroup bias in face recognition/evaluation (Kurzban et al., 2001; Lebrecht et al., 2009; Hehman et al., 2010) and affective evaluation (Van Bavel and Cunningham, 2009).

### Racial Bias in Neural Response for Pain Is Modulated by Minimal Group

To further investigate whether racial bias in neural response for pain is modulated by minimal group, we calculated the correlations between neural responses of the rAI (which is related to ingroup bias as we have discussed) and the ACC (which is related to racial bias as we have discussed) in the pain condition. We found a positive correlation in the contrasts of ingroup vs. outgroup, but not in the contrasts of Chinese vs. Western, between the ACC and rAI in the pain condition, indicating that racial bias can be modulated by ingroup bias in the pain condition. Our results are in line with a previous ERP study that racial empathic bias can be affected by minimal group (Sheng and Han, 2012).

We also calculated the correlations between neural responses of the rAI (which is related to ingroup bias as we discussed) and the ACC (which is related to racial bias) in the nopain condition. Interestingly, we found a positive correlation in the contrasts of Chinese vs. Western, but not in the contrasts of ingroup vs. outgroup, between the ACC and rAI in the nopain condition, indicating that ingroup bias modulates racial bias in the nopain condition. We also note that neural responses of the ACC were modulated by implicit racial bias differently between nopain and pain conditions, i.e., a positive correlation between racial contrasts of the ACC and D-index in the nopain condition, but a negative correlation in the pain condition. It is high unlikely that the increased activity in the nopain condition reflects higher empathy in participants with higher racial bias. Instead, these results suggest that different mechanisms are underlying nopain and pain conditions. Further detailed study may tackle this question. Nevertheless, these differences could lead to larger individual differences and make it harder to detect possible effects of ingroup bias on racial bias in previous studies (Contreras-Huerta et al., 2013, 2014).

### Limitations

Our study bears several limitations. First, there was a large discrepancy in the gender of our participants (only four males). Stereotypically, females are portrayed as more nurturing and empathetic (Christov-Moore et al., 2014) and self-report data consistently indicates greater empathy in women (Baez et al., 2017). Despite experimental and neuropsychological measures show no consistent sex effect on empathy in most studies (Baez et al., 2017) but not all (Yang et al., 2009), it is possible that our results may be affected by gender discrepancy and further study is needed to address this question. The second issue is that no significant interaction was found in the voxel-wise analysis of imaging results. We note that the effects of minimal grouping on racial bias are very weak. Thus, this study may lack the power to detect such small difference. Further study using more participants is desired to address this issue. The third one is related to frequently reporting extremely high correlations between measures of emotion and BOLD signals of brain, or “Voodoo correlations” (Vul et al., 2009). Note that in this kind of study, typically, a voxel-wise approach was used and voxels were selected because they correlated highly with the behavioral measure of interest. However, Vul et al. (2009) also argued that the “Voodoo correlations” can be avoided by an ROI approach in which ROI is selected “blind” to the correlations of BOLD signals with the behavioral measure, as in the present study. Moreover, a sample correlation based on 25 subjects has an approximate 95% confidence interval of ± 0.4 (Nichols and Poline, 2009). In our case, data from 29 participants were used for the correlation analysis. Thus, we believe that our results are robust and the possibility of “Voodoo correlation” is very limited.

## Conclusion

In this study, we reported that there are different neural correlates of racial bias and ingroup bias, and moreover, racial bias in neural response for pain is modulated by minimal group. Our findings indicate that even minimal group is sufficient to induce ingroup bias to reduce racial bias, especially in pain condition. Our findings provide important insights about racial bias, ingroup bias, as well as their dynamic relationship between nopain and pain conditions, and may have implications for understanding real-life social behaviors and provide a mechanism for a simple way to reduce racial bias.

## Ethics Statement

This study was carried out in accordance with the recommendations of the University of Committee on Human Research Protection of East China Normal University with written informed consent from all subjects. All subjects gave written informed consent in accordance with the Declaration of Helsinki.

## Author Contributions

ZW and HW designed the study, analyzed the data, and wrote the paper. MF designed the study and collected the data. FS and YH collected the data, analyzed the data, and wrote the paper.

## Conflict of Interest Statement

The authors declare that the research was conducted in the absence of any commercial or financial relationships that could be construed as a potential conflict of interest. The handling editor declared a past co-authorship with the authors ZW and YH.

## Footnotes

1 http://www.fil.ion.ucl.ac.uk/spm/
